# Developing bacterial resistance to antibiotics: a laboratory activity

**DOI:** 10.1099/acmi.0.001118.v3

**Published:** 2026-01-07

**Authors:** Maya Hagander, Claire DeWeese, Rachel Boyette, Paige Rudolf, Daniel R. Marous

**Affiliations:** 1 Department of Chemistry, Wittenberg University, Springﬁeld, OH 45504, USA

**Keywords:** antibiotic resistance, laboratory activity, science awareness

## Abstract

Bacterial resistance to antibiotics continues to be a major threat to human health. Agencies, such as the World Health Organization, have called for a multistep response, including increased educational training, both for professionals and the public at large, on this complex problem. Toward that end, we created a laboratory experience, ideally suited for undergraduates, where students observe the development of bacterial resistance over the course of a week. The procedure is conducted in a single container for simplicity and reliably generates resistant strains. Eightfold increases in resistance levels were commonly observed. Multiple variations to the standard method are included and explore the effects of antibiotic concentration and additivity. In performing the activity, students learn basic microbiology techniques, including growing bacterial cultures and determining MICs. Students are able to witness the relative ease with which bacteria can become resistant and then connect this concept to natural selection. The activity itself was created in partnership with undergraduate student researchers, a practice that is becoming more prevalent. Overall, the activity promotes understanding and awareness of antibiotic resistance, which is critically needed to combat this global threat.

**Impact statement**

We have created a laboratory experience, in collaboration with undergraduate researchers, where students observe the development of bacterial resistance to antibiotics and increase their awareness about this significant health threat.

## Data Summary

The authors confirm that all supporting data, code and protocols have been provided within the article.

## Introduction

In the foreword to the Centers for Disease Control’s 2019 report on Antibiotic Resistance Threats in the USA, the director urged the nation to stop referencing the impending post-antibiotic era; it has already arrived [1]. Indeed, the same report stressed that more than 2.8 million antibiotic-resistant infections occur annually in the USA, resulting in at least 35,000 deaths. Antibiotic use can also lead to *Clostridioides difficile* infection, which, in 2017, caused over 200,000 cases that required hospital care and nearly 13,000 deaths [1]. Both in the USA and internationally, bacterial resistance to antibiotics looms as a huge threat to health [2].

While antibiotics are staples of modern healthcare, their overuse and misuse in medicine and agriculture have exacerbated the spread of resistance. The abundance of antimicrobials in the environment encourages the selection of resistant bacterial populations. The resistant traits themselves are developed through multiple mechanisms [3]. In some cases, resistance is acquired through horizontal gene transfer [3,4], including a resistance gene on a plasmid that is shared between bacteria. However, a population of bacteria can also develop resistance without the need for foreign genetic material. When bacteria are treated with an antibiotic, a very small percentage of cells are impervious to the challenge, perhaps due to a beneficial mutation or gene duplication [4]. Upon treatment, the subset of resistant cells is selected due to a survival advantage. Several reviews address the mechanisms of antibiotic resistance in additional detail [3,5, 6].

To combat antibiotic resistance, a multifaceted response is needed, including the education of healthcare students as well as the general population, which has been recognized nationally [7] and internationally [8]. Educational efforts, especially in the classroom, would ideally address common misconceptions about antibiotic resistance, including (1) people, rather than bacteria, develop resistance to antibiotics and (2) bacteria undergo mutation to evade antibiotics [9]. The latter fallacy, in particular, stresses the importance of understanding natural selection in the context of antimicrobial resistance. While the point of emphasis likely varies with context, several educational approaches targeting diverse audiences have been developed and were recently reviewed by Marvasi and colleagues [2]. Examples from the review and elsewhere include: forum theatre plays [10], games/activities [9,11,13], an online multidisciplinary course [14], an ‘antibiotic prescribing etiquette’ course for medical students [15], a kinesthetic exercise [16], a serious computer-based game [17], course-based undergraduate research experiences (CUREs) [18,19] and a lab exercise [20]. A few of these are focused on undergraduates and involve student work in laboratories [18,20]. The CUREs allow college students to examine antibiotic (tetracycline) resistance in the environment by collecting and analysing soil samples [18,19]. In the aforementioned lab exercise, the students prepare a growth competition between sensitive and resistant bacteria, either with or without selective pressure (antibiotic) [20].

In our work described herein, we created a lab experience where students observe the development of resistance starting from sensitive strains. The exercise allows students to use live strains and witness the relative ease with which bacteria can become resistant. The experience was developed in ‘partnership’ with undergraduate students, where students themselves were deeply involved in the co-creation of teaching resources [21]. The concept of co-designing curricular materials with students has existed for many years [22] but is undergoing a resurgence recently and becoming more widespread [21,23,25]. Using ‘students as partners’ [26] has many benefits, including transforming the ways students think and practise [27], building favourable relationships between faculty and students [28] and making students more aware and confident in their engagement in the educational process [29]. The four undergraduates involved in the development of this lab experience are included as co-authors.

Given the techniques involved, our procedure is best suited for an undergraduate laboratory (student ages 18–22), though it would potentially work in a well-equipped high school setting as well. For our bacterial strain, we selected *Escherichia coli* ATCC 25922, which is relatively innocuous (biosafety level 1) and for which antibiotic susceptibility data are abundant [30]. We utilized two readily available antibiotics from distinct classes: ampicillin (beta-lactam) and kanamycin (aminoglycoside). There are known methods for evolving resistance in bacteria; however, they often involve a bioreactor [31,32] or the daily inoculation of fresh medium [33,35]. For time considerations, we developed a single-pot procedure that minimizes the effort required each day.

In addition to facilitating conversations to address the aforementioned misconceptions, the learning objectives for this activity include the following:

- To describe the antibiotic resistance threat
- To perform microbiology techniques, including growing bacterial cultures and determining MICs
- To observe the selection of bacterial resistance over the course of a week

## Methods

### Materials and equipment

*E. coli* bacterial strain ATCC 25922 (biosafety level 1) was used for all experiments and was purchased directly from the American Type Culture Collection. A similar strain would likely work as well, though the particular MICs may differ. The following reagents and equipment were used in the procedure: kanamycin monosulfate (Gold Biotechnology), ampicillin (sodium salt, Gold Biotechnology), BD Difco™ dehydrated culture media [Luria–Bertani (LB) broth, Miller], 13 mm × 100 mm culture tubes (Fisher Scientific), 50-ml Erlenmeyer flasks, shaking and non-shaking incubators (VWR 10L Shaking Incubator H1010, VWR Forced Air General Incubator 414005-124, respectively) and a spectrophotometer (Genesys 20, Thermo Spectronic).

### Hazards

Use PPE, including gloves and goggles, when handling bacterial cultures and antibiotics. PPE minimizes the risk of an allergic reaction associated with antibiotic exposure. Individuals with known allergies to aminoglycosides or beta-lactam antibiotics may want to avoid using that antibiotic. Students should be trained in using Bunsen burners for sterile technique. Cultures should be bleached or autoclaved (121 °C, ~60 min) when the procedure is completed. For bleaching cultures, add household bleach to 10% of the culture volume, and let the culture sit for at least 30 min before flushing down the drain with plenty of cold water.

### Selection of student researchers

The undergraduate students who participated in the development of this lab exercise applied to Wittenberg University’s Chemistry Summer Research Program (offered each summer) and were invited to work on the project based upon their stated individual interests. While the framework and goals of the exercise were provided, the students undertook the majority of the physical experiments and determination of the optimal conditions.

### Laboratory experience procedure

The students are assigned an antibiotic, kanamycin (Kan) or ampicillin (Amp) and are provided with the MIC for that antibiotic against the *E. coli* strain (25 µg ml^−1^ and 4–8 µg ml^−1^ for Kan and Amp, respectively). The students verify this MIC value at the end of the exercise. If time or supplies are limited, students could complete the work in pairs. The students are given a sterile 50-ml Erlenmeyer flask, an overnight *E. coli* culture, sterile LB medium and the antibiotics as solid powders. The students are directed to make stock solutions of their respective antibiotics (for example, 25 mg ml^−1^ for Kan and 1 mg ml^−1^ Amp; to avoid contamination, these stocks could be sterile-filtered). The students are instructed to make a 10 ml culture containing 50% of the MIC for their antibiotics (12.5 µg ml^−1^ and 2 µg ml^−1^ for Kan and Amp, respectively). The students are then directed to calculate the correct amount of antibiotic to add to 10 ml of LB medium. Since the volume of the antibiotic is so small compared to the volume of the medium, the volume of the antibiotic is usually ignored when determining the final culture volume. Using the overnight *E. coli* culture, the students make a diluted culture in a glass culture tube that has an optical density at 600 nm (OD_600_) of 0.1; this roughly corresponds to 10^8^ c.f.u. ml^−1^) [36]. The 10 ml of LB medium is inoculated with 100 µL of the diluted culture, giving a final inoculum of ~10^6^ c.f.u. ml^−1^. Once the medium, antibiotics and bacteria are added to the flasks, the cultures are placed in a non-shaking incubator (37 °C). On each of the next 3 days (days 2, 3 and 4), 5 ml of additional medium is added to the cultures along with enough antibiotic to double the total concentration of antibiotic (see Table 1), assuming no antibiotic degradation. The students are asked to calculate how much antibiotic to add each day (days 2, 3 and 4) to achieve the desired concentrations. These calculations are not necessarily trivial for the students, considering the volumes are changing and one must account for the previous additions. If the students have difficulty, they can be instructed to start with the total LB volume, calculate the total antibiotic volume needed and then subtract the volume of antibiotic previously added. The volumes of LB medium and example volumes of Kan or Amp that are added each day can be seen in Table 2.

**Table 1.** Total amount of LB medium and antibiotics in cultures

	LB (ml)	Kan (μg ml^−1^)	Amp (μg ml^−1^)
Day 1	10	12.5	2
Day 2	15	25	4
Day 3	20	50	8
Day 4	25	100	16

**Table 2.** Volume of LB medium and antibiotics added to cultures

	LB (ml)	25 mg ml^−1^ Kan (μl)	1 mg ml^−1^ Amp (μl)
Day 1	10	5	20
Day 2	5	10	40
Day 3	5	25	100
Day 4	5	60	240

**Fig. 1.**
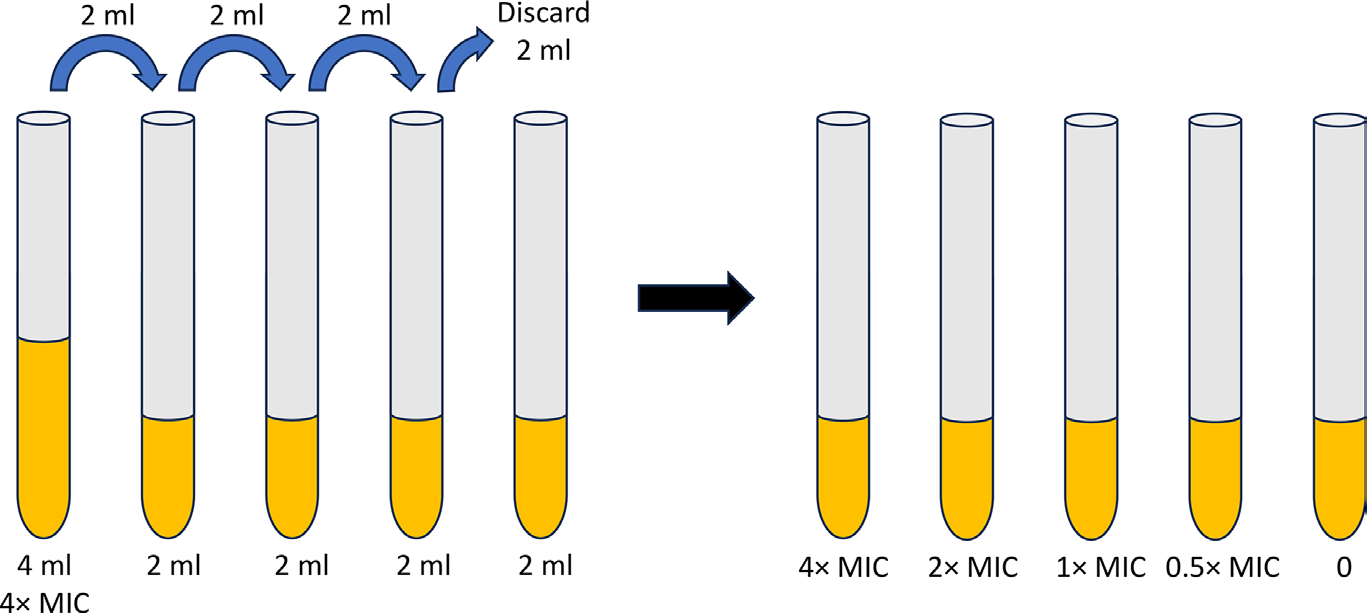
;Illustration of serial dilution technique for MIC testing. Sequential transfer of 2 ml solution creates a concentration gradient across five tubes. Final tubes contain decreasing concentrations labelled as 4x, 2x, 1x, 0.5x and 0 MIC.

On day 5, no additions are made to the cultures, and they are removed from the incubator and placed on the benchtop at room temperature over the weekend. This step could be completed by the instructor. In the event that Friday is earlier than day 5, no additions are made on Friday, the cultures are left at room temperature and the next addition is resumed on Monday. During the lab period in the following week (day 8), the students complete an MIC test for their antibiotic-exposed strain as well as the wild-type *E. coli* strain. Each MIC test requires at least five sterile culture tubes; 2 ml of LB medium is pipetted into four of these tubes, while the first tube receives 4 ml of medium. Antibiotic is added to the 4-ml tube to achieve a final concentration of 4× MIC (100 µg ml^−1^ for Kan or 16 µg ml^−1^ for Amp). Two millilitres of the antibiotic-containing medium is serially diluted into three more culture tubes to achieve concentrations of 2×, 1× and 0.5× MIC, respectively (Fig. 1). Note the final 2-ml culture tube, corresponding to 0 µg ml^−1^ antibiotic, does not receive a serial dilution. All the culture tubes should have 2 millilitres of medium when the serial dilution is completed. The students again make a diluted culture with an OD_600_ equal to 0.1 and use this to inoculate the five culture tubes (20 µl inoculum, giving a final concentration of ~10^6^ c.f.u. ml^−1^).

The covered culture tubes are placed in a shaking incubator (37 °C, 250 r.p.m.) overnight. *E. coli* growth is assessed the next day visually (cloudy vs. clear) or using a spectrophotometer. If a spectrophotometer is used, an OD_600_ greater than 0.05 is used to indicate growth [35]. The lowest antibiotic concentration tube that is clear gives the MIC. For convenience, the highest concentration that can be tolerated is referred to as the highest tolerated concentration (HTC). The tolerated concentrations are compared between the wild-type and antibiotic-exposed strains. A summary of the procedure is given in Table 3.

**Table 3.** Summary of procedure

Day	Task
0	Instructor prepares medium and starts an overnight *E. coli* culture
1	Students start their cultures and add antibiotics
2–4	Students add medium and antibiotics to cultures
5	No additions are made to cultures, and they are left at room temperature
7	The instructor starts an overnight wild-type *E. coli* culture
8	Students complete MIC tests for antibiotic-exposed and wild-type strains
9	Students determine the results of MIC tests and dispose of cultures

### Variations

Several variations on the procedure outlined above were also explored with student researchers. In one variation, no antibiotic was added to the wild-type culture, only additional LB medium on days 2–4. In the second variation, the antibiotic concentration on day 2 was increased eightfold, instead of the usual twofold, to reach the final antibiotic concentration for each antibiotic (see Table 1). In this case, the MIC tests were conducted on day 3, rather than day 8. In the third variation, an *E. coli* culture was given daily additions of both Kan and Amp (following Table 2), instead of just one antibiotic.

## Results

By exposing the *E. coli* strains to increasing concentrations of antibiotics over the course of a week, the cultures are under selective pressure to evolve resistance to the antibiotic. The student researchers, named as co-authors, frequently observed the development of resistance in the antibiotic-exposed strains. With the Kan-treated strains, they were usually resistant to 50 µg ml^−1^ (2/10 strains) or 100 µg ml^−1^ Kan (7/10 strains). Assuming no Kan degraded in the cultures, 100 µg ml^−1^ is the maximum possible concentration of Kan to which the cultures were exposed. There was one trial, where no additional resistance was observed (HTC=12.5 µg ml^−1^, the wild-type level). Often, the Amp-treated strains were resistant to at least 8 µg ml^−1^ Amp (6/8 strains). If no Amp degraded over the course of a week, theoretically, the cultures contained 16 µg ml^−1^ Amp. There were also resistance levels as high as 32 µg ml^−1^ Amp and as low as 4 µg ml^−1^ Amp, the latter of which is comparable to wild-type resistance levels. The 32 µg ml^−1^ Amp resistance highlights that a particular resistance mechanism may confer resistance to an antibiotic concentration beyond that to which the culture was exposed. A summary of the resistance levels of the Kan and Amp-treated strains is shown in Table 4.

**Table 4.** Resistance levels of antibiotic-exposed strains

Kan (μg ml^−1^) (*n*=10)	Amp (μg ml^−1^) (*n*=8)
12.5 (1)	4 (2)
50 (2)	8 (5)
100 (7)	32 (1)

**Fig. 2.**
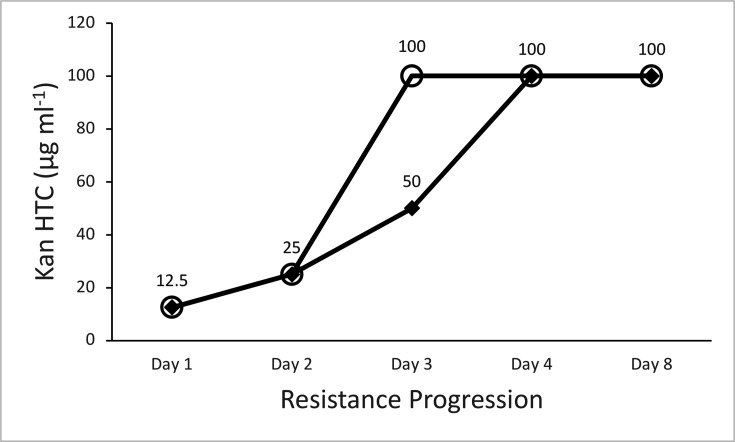
Line graph showing kanamycin HTC values increasing from 12.5 to 100 μg ml^−1^ during resistance progression. Two cultures show different rates, with circle-marked culture reaching maximum at day 3 and diamond-marked culture at day 4.

To determine how the resistance level varied throughout the course of the procedure, two additional cultures with Kan exposure were grown, where a daily MIC test was conducted prior to any LB or antibiotic additions (Fig. 2).

While both cultures achieved the same final HTC (100 µg ml^−1^), one culture reached that level on day 3 and the other a day later. Interestingly, the final Kan addition, which brought the total Kan to ~100 µg ml^−1^, did not occur until after the MIC test on day 4, at which point both cultures were already resistant to 100 µg ml^−1^ Kan. Thus, again, we observed the HTC exceeding the exposure concentration. In these two trials, the day 4 antibiotic addition did not appear to confer additional resistance to 200 µg ml^−1^ Kan.

Though not delineated in the standard procedure above, the student researchers also investigated whether resistant strains were cross-resistant to the antibiotic to which they were not exposed. For example, a strain resistant to 8 µg ml^−1^ Amp had a Kan HTC of 25 µg ml^−1^ (twice the wild-type HTC) and a strain resistant to 100 µg ml^−1^ Kan had an Amp HTC of 4 µg ml^−1^ (wild-type HTC). As Kan and Amp are in distinct antibiotic classes, it is conceivable that resistance to one does not guarantee resistance to the other; it depends on the mechanism of resistance, which may vary between strains.

In a trial where the wild-type strain was given additional LB throughout the week but not exposed to antibiotics, the final HTCs, 12.5 and 4 µg ml^−1^ for Kan and Amp, respectively, were comparable to the wild-type resistance levels. Because no antibiotic is present, the culture lacks selective pressure to evolve resistance. In other trials, strains were started with normal antibiotic concentrations (12.5 µg ml^−1^ and 2 µg ml^−1^ for Kan and Amp, respectively), but on day 2, the concentrations were increased eightfold (to 100 µg ml^−1^ and 16 µg ml^−1^ for Kan and Amp, respectively). It was possible that this relatively large increase would kill the cultures; however, this was not the case. The final HTCs from three trials with each antibiotic can be seen in Table 5. Overall, while the cultures were viable, the HTCs were often lower than when the antibiotic concentration was increased more gradually.

**Table 5.** HTCs of strains exposed to an eightfold antibiotic increase on day 2

Trial	Kan-exposed (μg ml^−1^)	Amp-exposed (μg ml^−1^)
1	12.5	4
2	50	4
3	25	8

In a final variation, the student researchers gave the culture doses of both Kan and Amp simultaneously. This culture showed little growth and in a final MIC test, even the no antibiotic control did not grow, suggesting the culture was not viable. This variation is useful in highlighting to students how non-lethal doses of two antibiotics can be additive when combined.

## Discussion

Overall, a procedure has been developed that demonstrates the development of bacterial resistance to antibiotics. The procedure requires students in a course to participate on consecutive days, though the majority of the student effort is concentrated on days 1 and 8 (presumably the lab day for a typical undergraduate course). For the other days, lab partners could alternate when they complete the additions or an instructor or teaching assistant could perform the required steps. In the absence of an autoclave, sterile liquid medium could be purchased. If a spectrophotometer is not available at an institution, visual appearance (cloudy vs. clear) could be used to determine MICs, though a visual standard (McFarland) would be needed to generate cultures with an OD_600_ equal to 0.1. The student researchers also explored whether a shaking incubator was strictly necessary for the MIC cultures. Without shaking (but still incubation), the OD_600_ tended to be lower at a given antibiotic concentration. The MICs were sometimes lower (~twofold) without shaking, so all MIC tests should be conducted under the same conditions. For the Erlenmeyer flask cultures, however, the student researchers found the development of resistance was more reliable if the flasks were incubated but not shaken. Perhaps, the lack of shaking allows the bacteria to grow on the surface of the flask, similar to biofilms, which are known to facilitate antibiotic resistance [37]. Additionally, without shaking, the oxygen levels may be lower, impacting resistance development. For example, it has been shown that MICs for kanamycin and ampicillin, as well as other antibiotics, are increased under low oxygen conditions [38]. Moreover, the student researchers investigated different starting and addition volumes for the Erlenmeyer flasks as well as different weekend conditions (37 °C, room temperature or refrigeration). In the end, the 10 ml starting culture with 5 ml additions and a room temperature weekend incubation proved to be relatively straightforward to execute and effective at allowing the cultures to become resistant.

As noted in the results, the procedure reliably generates resistant strains, along with some natural variability. Generally, the student researchers observed more consistency with Kan than Amp in terms of MIC values and getting high resistance levels. For example, depending on the trial, the wild-type MIC for Amp ranged between 4 and 8 µg ml^−1^. Some variability can occur with broth MIC testing, which is considered reproducible within a twofold dilution [39]. If a single value needs to be reported, the MIC can be recorded as the mode of triplicate testing [40]. For this activity, it may be useful for students in a course to see the entire class data set to observe any ranges in MIC values. While the Kan-treated strains were frequently resistant to approximately the highest concentration to which they were exposed, this appeared to be less common with the Amp-treated strains. However, the effective Amp concentration could be lower than expected as it may degrade in the culture medium over time due to its chemically unstable beta-lactam ring [41].

If time and resources allow, the variations to the standard procedure could provide helpful comparisons for students in a course. While the no-antibiotics and both-antibiotics variations behaved predictably, the eightfold jump variation displayed a range of outcomes. The generally lower HTCs seen with the eightfold jump are perhaps expected as these strains had less time and fewer generations to develop resistance to approximately the same final antibiotic concentration. Collectively, the variations allow for more in-depth discussion about bacterial resistance to antibiotics.

Our lab experience was developed in collaboration with student researchers during the summer, a model that others have used [25]. This arrangement worked well, as the students had fewer academic obligations than are present during the semester. However, a case has been proposed for using ‘students as partners’ in a whole-class context as well [28]. Such an arrangement is inherently more inclusive and fosters positive relationships between the instructor and a larger group of students, but is challenging to implement due to time constraints and large class sizes [28]. Regardless of the number of students, partnership practices in the literature have been grouped into four overlapping categories, including (1) subject-based research and inquiry, (2) scholarship of teaching and learning, (3) curriculum design and pedagogic consultancy and (4) learning, teaching and assessment [26]. The present study developed a pedagogical activity on the subject of bacterial resistance and thus most closely aligns with categories 3 and 1. In a literature review of 65 scholarly works, more than half of the studies involved category 3 (54%), but only 12% mapped to category 1 [26]. While subject-based research may be relatively rare in the ‘students as partners’ arena, it seems to be a natural fit in the sciences. By including students in the development of this laboratory activity, several outcomes were positively impacted. First, the procedure itself was improved. For example, Table 2 and Fig. 1 were originally drafted by students out of a need to clarify the methodology for themselves and others. The room temperature weekend incubation of the cultures was also the direct result of a student’s idea. Secondly, there was an enhanced relationship built between the students and the faculty member. Finally, the joint project improved career prospects and networking for both the students and the faculty member, particularly as the project was shared at conferences. All of these outcomes have been previously observed in studies using ‘students as partners’ [26].

The present study focused on developing the methodology to reliably demonstrate bacterial resistance; however, it did not measure student learning gains from completing the activity. Future work could implement the activity along with a pre-/post-assessment to ascertain the degree to which students increased their understanding of antimicrobial resistance and whether common misconceptions were corrected.

## Conclusion

In summary, this lab activity allows students in a course to become familiar with the antibiotic resistance threat, perform microbiology techniques and witness the evolution of resistance in a single-pot procedure. These students observe that resistance occurs in bacteria, rather than in people, and can connect this idea to natural selection. As the procedure involves students physically growing and assessing resistance strains, it promotes active learning on antimicrobial resistance. Collectively, the activity can be used to improve awareness and interest in a clinically relevant issue.

## Abbreviations

Amp: ampicillin
c.f.u.: colony-forming unit
CUREs: course-based undergraduate research experiences
HTC: highest tolerated concentration
Kan: kanamycin
LB: Luria–Bertani
